# Species-specific sensitivity to intracerebroventricular streptozotocin in rats and mice highlights pathways and proteins relevant to Alzheimer’s disease

**DOI:** 10.1007/s00702-025-02952-w

**Published:** 2025-06-01

**Authors:** Jan Homolak, Pavel Markovic, Davor Virag, Ana Knezovic, Jelena Osmanovic Barilar, Andrija Loncar, Melita Salkovic-Petrisic, Ana Babic Perhoc

**Affiliations:** 1 Department of Pharmacology, University of Zagreb School of Medicine, Zagreb, Croatia; 2 Croatian Institute for Brain Research, University of Zagreb School of Medicine, Zagreb, Croatia; 3 Interfaculty Institute for Microbiology and Infection Medicine Tübingen, University of Tübingen, Tübingen, Germany; 4 Cluster of Excellence EXC 2124 Controlling Microbes to Fight Infections, University of Tübingen, Tübingen, Germany; 5 M3-Research Center for Malignome, Metabolome and Microbiome, University of Tübingen, Tübingen, Germany; 6 Neurodegenerative Diseases Research Group, Vall d’Hebron Research Institute (VHIR)-Network Center for Biomedical Research in Neurodegenerative Diseases (CIBERNED), Barcelona, Spain; 7 Division for Vascular Neurology and Neurodegenerative Diseases, Department of Neurology, UHC Sisters of Mercy, Zagreb, Croatia

**Keywords:** Streptozotocin, Alzheimer’s disease, Animal model, Rat, Mouse

## Abstract

For over three decades, intracerebroventricular streptozotocin (STZ-icv) has been a key non-transgenic rodent model of sporadic Alzheimer’s disease. Traditionally used in rats, its use in mice is growing due to practical advantages. While factors like strain, sex, vehicle, age, and dosage are well studied in STZ-induced diabetes models, their impact on STZ-icv-related cognitive deficits remains poorly understood. To address this gap, we examine species-specific sensitivity to STZ-icv in two widely used rodent strains (Wistar Han rats and C57BL/6 mice) motivated by the finding that, despite reliably inducing cognitive deficits in rats for over 30 years in our lab, the same STZ-icv procedure yields inconsistent results in C57BL/6 mice. To explore this phenomenon, we first constructed a series of allometric models to assess how different STZ-icv concentrations affect predicted exposure in rats and mice. Next, we provide behavioral and immunohistochemical evidence that C57BL/6 mice exhibit resistance to STZ doses that should achieve sufficient neuronal exposure. Finally, we perform an exploratory multiomic analysis, examining differentially expressed proteins in the hippocampus and prefrontal cortex of STZ-icv-treated rats that also show differential expression between Wistar Han rats and C57BL/6 mice, followed by an investigation of these proteins across independent Alzheimer’s disease cohorts. Through this analysis, we identify three understudied proteins—Tagln2, Slc25a3, and Pafah1b3—that may contribute to the differential sensitivity of Wistar Han rats and C57BL/6 mice to STZ-icv and may play a role in the development and progression of Alzheimer’s disease.

**Supplementary Information:**

The online version contains supplementary material available at 10.1007/s00702-025-02952-w.

## Introduction

Alzheimer’s disease (AD) is the most prevalent neurodegenerative disorder, with a rising global incidence. However, the development of effective treatments remains challenging due to an incomplete understanding of its etiopathogenesis. Preclinical animal models of AD are therefore crucial for developing and testing new therapeutic strategies, particularly for sporadic AD (sAD), which accounts for over 95% of cases. For more than three decades, intracerebroventricular administration of streptozotocin (STZ-icv) has been widely used to model sAD in rodents, making it one of the most commonly employed non-transgenic rodent models of AD. Since its introduction by the pioneers of brain insulin resistance research, Hoyer (Mayer et al. 1990) and Salkovic-Petrisic (Lacković and Salković 1990), the STZ-icv model has traditionally been used in rats to study disease pathophysiology (Homolak et al. 2023; Homolak et al. 2024; Homolak et al. 2021; Homolak et al. 2022; Osmanovic Barilar et al. 2022; Xing et al. 2025; Alves et al. 2023; Dunacka et al. 2024) and evaluate pharmacological compounds (Salkovic-Petrisic et al. 2013; Osmanović Barilar et al. 2023; Knezovic et al. 2023; Fine et al. 2024; Dahiya et al. 2025; Zidan et al. 2024; Naeem et al. 2024; Jain et al. 2025; Kaur et al. 2024) and promising non-pharmacological interventions for sAD (Knezovic et al. 2018; Salkovic-Petrisic et al. 2014; Zhang et al. 2025; Soleimani et al. 2024) by a number of groups. However, the advantages of using smaller rodents, particularly mice—such as reduced costs, smaller amounts of compounds required for testing, ease of handling, and access to extensive genetic resources—have led to an increasing number of laboratories adopting the STZ-icv modelling approach in mice (e.g. (Xing et al. 2025; Jain et al. 2025; Wong-Guerra et al. 2025; Khamies et al. 2024)).

Interestingly, while there is a wealth of literature on how factors such as STZ vehicle, preparation technique, dose, and administration protocol as well as animal species, strain, sex, and age, influence rodent susceptibility to STZ in diabetes models (Ghasemi and Jeddi 2023; Deeds et al. 2011), there is still limited research on how these characteristics might affect susceptibility to STZ-icv-induced cognitive deficits (Homolak et al. 2021). In this study, we address the gap in understanding by examining species-specific sensitivity to STZ-icv in the two most commonly used strains for modelling sAD—male Wistar Han rats and male C57BL/6 mice—motivated by the observation that applying the same procedure, which has reliably induced cognitive deficits in rats for over 30 years in our lab (Homolak et al. 2021; Salkovic-Petrisic and Kostrzewa 2021), produces less consistent results in C57BL/6 mice. We begin by examining allometric scaling to understand how different doses of STZ administered to the lateral brain ventricles translate to brain STZ exposure, a crucial parameter given that many laboratories adjust STZ-icv doses based on animal weight under the incorrect assumption of isometric body-brain scaling in rodents. Next, we provide evidence that, similar to STZ-induced diabetes (Cardinal et al. 1998), mice appear less susceptible to STZ-induced cognitive deficits. Finally, we adopt a bioinformatics approach to identify proteins and signaling pathways that may account for species-specific differences in sensitivity to STZ-icv, analyzing these findings in the context of consistent proteomic changes observed in human AD patient cohorts.

## Materials and methods

### Animals

#### Rats

Rats used in EXP R1, R2, R3, R4, R5, and R6 were male Wistar Han rats bred and held at the University of Zagreb School of Medicine (UZSM) licensed animal facility at the Department of Pharmacology (HR-POK-007, Zagreb, Croatia). Rats were housed in standardized conditions, in groups of 2–3 rats per cage, with food and water ad libitum, controlled temperature (21–23 °C) and humidity (40–70%), and a 12 h-light/12 h-dark cycle. In all 6 experiments, rats underwent STZ-icv administration at 3-months-old.

#### Mice

In EXP M1, C57BL/6 male mice aged 10 weeks were purchased from Charles River Laboratories (Sulzfeld, Germany). After arrival at the licensed animal facility at the UZSM Croatian Institute for Brain Research (CIBR; HR-POK-006, Zagreb, Croatia), mice were kept in a 14-day-quarantine and later moved to a ventilated cabinet with temperature set to 23° and humidity at 50%. Mice were kept in cages in groups of 6, fed with standardized food pellets and water ad libitum, with a 12 h-light/12 h-dark circadian cycle. Similarly, in EXP M2, M3 and M5 mice were kept in the same conditions but were not purchased; animals of the same strain (C57BL/6) were bred at CIBR and moved to the above referenced ventilated cabinet at 6 weeks of age. M4 mice were bred and kept at the UZSM Department of Pharmacology licensed facility (HR-POK-007, Zagreb, Croatia). M2 and M4 mice were all male, while M3 and M5 mice were all female. In all five mice experiments, the animals entered the experiment and underwent STZ-icv or methylene blue (MB)-icv administration at 3-months-old.

To better understand the differences in sensitivity to STZ-icv between Wistar Han rats and C57BL/6 mice, we utilized data from our dataset of behavioral indicators from previous experiments, along with samples from our tissue bank. This approach aligns with the principles of the 3Rs, as we did not conduct additional animal experiments but instead leveraged existing data to address our research questions. Although minor differences in experimental design were present between studies (e.g., both the CTR and STZ groups received vehicle treatment), these variations had no impact on the results. An overview of the experiments used in the study is provided in Table 1.

**Table 1:** List of experiments in the study

Experiment	Species (sex)	STZ dose	Duration	Cognitive deficits	Conclusion
EXP R1	Wistar Han rat (male)	0.3 mg/kg 1 mg/kg 3 mg/kg	4 weeks	Yes	↓cognitive performance 4 weeks after STZ-icv
EXP R2	Wistar Han rat (male)	3 mg/kg	4 weeks	Yes	
EXP R3	Wistar Han rat (male)	3 mg/kg	12 weeks	Yes	↓cognitive performance 12 weeks after STZ-icv
EXP R4	Wistar Han rat (male)	3 mg/kg	12 weeks	Yes	
EXP R5	Wistar Han rat (male)	1.5 mg/kg	15 min-24 h	NA	↑GFAP (acute)
EXP R6	Wistar Han rat (male)	3 mg/kg	4 weeks	Yes	↑GFAP (chronic)
EXP M1	C57BL/6 mouse (male)	6 mg/kg	4 weeks	No	= cognitive performance 4 weeks after STZ-icv = GFAP (chronic)
EXP M2	C57BL/6 mouse (male)	6 mg/kg	4 weeks	No	= cognitive performance 4 weeks after STZ-icv
EXP M3	C57BL/6 mouse (female)	10 mg/kg	4 weeks	Mild	↓cognitive performance (mild) 4 weeks after STZ-icv
EXP M4	C57BL/6 mouse (male)	1.5 mg/kg	15 min–24 h	NA	= GFAP (acute)
EXP M5	C57BL/6 mouse (female)	NA	15 min–24 h	NA	adequate icv distribution to lateral ventricles
Additional data obtained by other groups used in the study					
Fine et al. (2024)	Long-Evans rat (male)	4.5 mg/kg	6 weeks	Yes	Differential transcriptome
Da Silva et al. (2024)	Wistar Han rat (male)	3 mg/kg	2 weeks	NA	Differential proteome

*NA* not assessed, *GFAP* glial fibrillary acidic protein

### Ethics

All animal procedures were performed by certified personnel and executed in compliance with present institutional, national and international guidelines on the management of experimental animals for scientific purposes. The conducted experiments were approved by the national regulatory body, Croatian Ministry of Agriculture, and the UZSM Ethical Committee (license numbers EP 384/2023, 525–10/0255-15-5, UP/I-322-01/11-01/10, 04-1343-2006, 380-59-10106-14-55/83-641-01/14-02/01, EP 186/2018-380-59-10106-18-111/173).

### STZ administration

STZ (Sigma-Aldrich, Taufkirchen, Germany) administration was performed across all 4 experiments by applying the same methodology initially described by Noble et al. (Noble et al. 1967), as published before by our research group (Knezovic et al. 2018; Knezovic et al. 2015; Grünblatt et al. 2007; Barilar et al. 2015). Animals aged 3 months were weighed and anesthetized using intraperitoneal applications of ketamine (50 mg/kg for rats, 100 mg/kg for mice) and xylazine (5 mg/kg for rats, 10 mg/kg for mice). After incising the skin, the skull was drilled using a specialized dentist drill, making two 1 mm holes, located above the lateral ventricles (rat coordinates: A/P: −1.5 mm; M/L: ± 1.5 mm; D/V: + 4 mm, mice coordinates: A/P: − 0.5 mm; M/L: ± 1.0 mm; D/V: + 2.5 mm). STZ was given bilaterally, into each lateral ventricle (2 µL/ventricle for rats; 1 µL/ventricle for mice) in a total dose of either 1.5, 3, 6 or 10 mg/kg (dissolved in 0.05 M citrate buffer, pH 4.5, divided equally in two administrations 48 h apart except in acute experiments (EXP R5 and EXP M4) where tissue analysis was conducted in specified time points after the first STZ-icv administration), using a Hamilton microliter syringe with a stopper, without stereotaxic control (Homolak et al. 2021). Controls (CTR) underwent the same procedure and received an equal volume vehicle icv (0.05 M citrate buffer, pH 4.5). Incision sites were sutured and the animals monitored during anesthesia recovery.

### Methylene blue administration

MB administration was performed in 3 female mice aged 3 months to validate intracerebroventricular administration procedure in mice using a standard procedure (Taylor et al. 2021; Redrobe et al. 2002). MB was prepared as a 1% solution in PBS. The procedure followed the same protocol as STZ-icv administration described above; mice were anesthetized with a combination of ketamine and xylazine (100/10 mg/kg, respectively), their skin incised on the skull, two holes drilled above the lateral ventricles, and 1 µL of MB solution was administered bilaterally following the above specified coordinates for STZ-icv in mice. Euthanasia by cervical dislocation was performed shortly after the administration, before the effects of anesthesia wore off. Brains were quickly removed and sectioned for analysis.

### Behavioral testing

Passive avoidance and/or T-maze were performed 4 weeks after STZ-icv administration in EXP M1, M2, M3, R1 and R2, and 3 months after the procedure in R3 and R4.

### Passive avoidance test (PA)

Conditional fear memory was examined using a step-through passive avoidance apparatus (Ugo Basile, Comerio, Italy) which exploits the rodents’ natural preference for dark environments (Walters and Abel 1971). During the test, animals learn to avoid stepping through a door from a brightly lit compartment, to an apparently safer, but previously punishment-related, dark compartment. The latency to avoid crossing into the darker compartment is measured and used as an index of the ability to avoid and allows retention memory to be evaluated. The test is performed during 3 days, with habituation as day 1 (no foot shock, i.e. pre-shock latency), electric shock delivery on day 2 (for rats, 0.3–0.5 mA, depending on the rat weight, duration 2 s; for mice, 0.2 mA, duration 2 s) and latency measurement on day 3. The time required to step through the door and enter the dark compartment (latency) is measured with a cut-off time of 300 s. The apparatus is cleaned between each animal using 30% ethanol.

### T maze

T-maze cognitive testing was performed using a spontaneous alteration protocol as described by d’Isa et al. (2021). The test was carried out in a T-shaped apparatus, custom made for rodent behavioral investigation. Briefly, each mouse was placed at the starting point (south-facing arm of the T-shaped apparatus) of the maze for the initial (T0) trial, facing the wall of the maze. Once the animal faces the center of the maze, time measurement is initiated, and the animal is allowed to explore the maze freely for 60 s. Once the animal chooses on of the opposing arms, the choice is annotated, and a removable door is placed behind the animal, confining it in the arm for 30 s. If the animal does not choose an arm within the designated 60 s, it is removed from the maze and re-tested after at least 30 min. After the initial 30 s arm confinement, animals are subjected to the same procedure for 6 more times (T1–T6), with a cut-off time for exploring set to 180 s. Every choice of arm is recorded, and after the final (T6) trial, the animal is returned to its home-cage. The maze is cleaned with 30% ethanol between each animal. To minimize stressful response, tail-picking is avoided, and the mice are carried from the home-cage to the maze via a cardboard tunnel used in environment enrichment. The measured percentage of alternation across the test trials (different choice of arm after each trial) is calculated as an index of working memory.

### Statistical analysis of behavioral readouts

Behavioral differences between control and STZ-icv treated animals in both the passive avoidance task and the T-maze were evaluated using linear models, with continuous behavioral outcomes as dependent variables and treatment, experiment, and their interaction as fixed effects. Model assumptions were verified through visual inspection of QQ plots. Estimated marginal means and p-values were computed using the *emmeans* package.

### Immunohistochemistry

Astrogliotic changes were analyzed using animal tissue from the tissue bank of the Laboratory for Molecular Neuropharmacology. All tissues were obtained from animals euthanized under deep anesthesia in previous experiments, for which ethical approvals had been obtained. The stored tissue had undergone a standardized procedure: first, blood was cleared via transcardial perfusion with 4% paraformaldehyde (PFA) at pH 7.4, followed by in vitro equilibration in PFA at 4 °C with increasing sucrose concentrations (up to 30%) for cryopreservation and long-term storage at −80 °C. The tissue was then embedded in Tissue-Tek O.C.T. (Sakura, Japan), sectioned using a Leica CM1850 cryostat (Leica Biosystems, Germany), blocked with a buffer containing 5% normal goat serum in 0.25% Triton X-100 in PBS, and incubated overnight at 4 °C with an anti-glial fibrillary acidic protein (GFAP) antibody (Sigma-Aldrich, USA). After washing in PBS, the sections were incubated with appropriate Alexa fluorophore-conjugated secondary antibodies (Cell Signaling Technologies, USA). Visualization was performed using an Olympus BX51 microscope and CellSens Dimensions software and Olympus FV3000RS Confocal Microscope.

### Allometric scaling models

STZ exposure models were designed using estimates of rodent brain weights and the numbers of neuronal and non-neuronal cells reported by Herculano-Houzel et al. (Herculano-Houzel et al. 2006). Standard deviation values from reported rodent brain measurements were considered to construct the intervals. Molarity calculations were based on in-house data on STZ preparation methods, following protocols established in the lab for over 30 years to model sAD in rodents, as described elsewhere (Homolak et al. 2021; Salkovic-Petrisic and Kostrzewa 2021). Specifically, 2 µL of STZ solution per ventricle was used for rats, and 1 µL for mice (Homolak et al. 2021; Salkovic-Petrisic and Kostrzewa 2021).

### Identification of relevant signaling pathways

To identify key differences in signaling pathways targeted by STZ-icv in Wistar Han rats and C57BL/6 mice, we used the following approach. First, we extracted brain gene expression data for male Wistar Han rats and male C57BL/6 mice from an RNA sequencing atlas of gene expression in normal mouse and rat tissues (Söllner et al. 2017). This approach minimized bias, as tissues from each species were processed using identical sample preparation methods, sequencing protocols, and bioinformatics pipelines (as described in (Söllner et al. 2017)). Homology information for mouse and rat genes was obtained from Ensembl (v84; in line with the approach described by Söllner et al.) using biomaRt, and species comparisons were limited to one-to-one orthologs. To ensure comparability, transcripts per million (TPM) were used to account for sequencing depth and transcriptome composition, given the similar complexity of the transcriptomes. Genes differentially expressed between rats and mice (log2 fold change < −1 or > 1) were then compared with differentially abundant proteins in the rat hippocampus (HPC) and prefrontal cortex (PFC) following STZ-icv treatment (3 mg/kg) in comparison to respective controls. For STZ-icv-treated rats, differential expression was defined as log2 fold change < −1 or > 1 with a p-value < 0.05. Analyses for PFC and HPC were conducted separately to identify genes with log2 fold changes < −1 or > 1 in both interspecies comparisons (Wistar Han rats vs. C57BL/6 mice) and in comparisons between STZ-icv-treated and CTR animals. Genes were classified based on confidence levels, with high confidence requiring a false discovery rate (FDR)-corrected p-value < 0.05 and low confidence defined by large effect sizes but an FDR-corrected p-value > 0.05. Proteins identified as hits in either brain region were pooled, deduplicated, and subjected to protein interaction network analysis using STRING (Szklarczyk et al. 2023). Functional enrichments were explored through local network clustering and Reactome pathway analysis. The results were further queried with additional node layers to identify key enriched pathways, which were classified based on strength (measuring the enrichment effect relative to random networks), FDR-corrected p-value, and signal (a weighted harmonic mean of the observed/expected ratio and −log(FDR)). Next, we used the dataset of STZ-relevant proteins differentially expressed in rats and mice to query the NeuroPro database, which contains extensive proteomic data from human AD cohorts (Askenazi et al. 2023). Our focus was on proteins identified in our analysis that showed consistent patterns of differential expression in at least five independent human studies, aligning with the direction of changes observed in the STZ-icv model. To further evaluate the significance of the identified proteins, we analyzed raw RNA seq data from Fine et al., obtained from the Gene Expression Omnibus database (CTR: GSM8630177, GSM8630178, GSM8630179; STZ: GSM8630180, GSM8630181, GSM8630182) (Fine et al. 2024). We performed differential expression analysis using DESeq2 (Love et al. 2014) median-of-ratios normalization to account for sequencing depth and transcriptome composition. For statistical testing, we used DESeq2 negative binomial generalized linear model with mean-based dispersion estimation to handle potential overdispersion. Log2 fold change were calculated using empirical Bayes shrinkage, followed by Wald tests to assess differential expression significance. P-values were adjusted via Benjamini–Hochberg FDR correction. We calculated TPM values, following a method similar to that used in the RNAseq atlas for mice and rats, to enable comparison with this approach and to rule out potential biases introduced during data processing. Next, we integrated the proteomic and transcriptomic datasets to identify common genes and proteins that differed in abundance between CTR and STZ-icv groups, as well as between mice and rats, allowing us to assess whether changes in the identified proteins corresponded with alterations in their transcriptional expression profiles. Furthermore, to gain deeper insights, we applied the same analytical approach used for proteomic data from da Silva et al. (Silva et al. 2024) to the RNAseq dataset from Fine et al. (Fine et al. 2024). This helped identify pathways and genes that may mediate the differential sensitivity of rats and mice to STZ-icv, even in cases where proteomic-level changes were subtle and would have otherwise been overlooked. Protein and gene interaction networks obtained by both approaches were analyzed using geneMANIA (Franz et al. 2018).

## Results

### Isometric dosing risks subexposure in the STZ-icv AD mouse model, according to allometric scaling

**Fig. 1:**
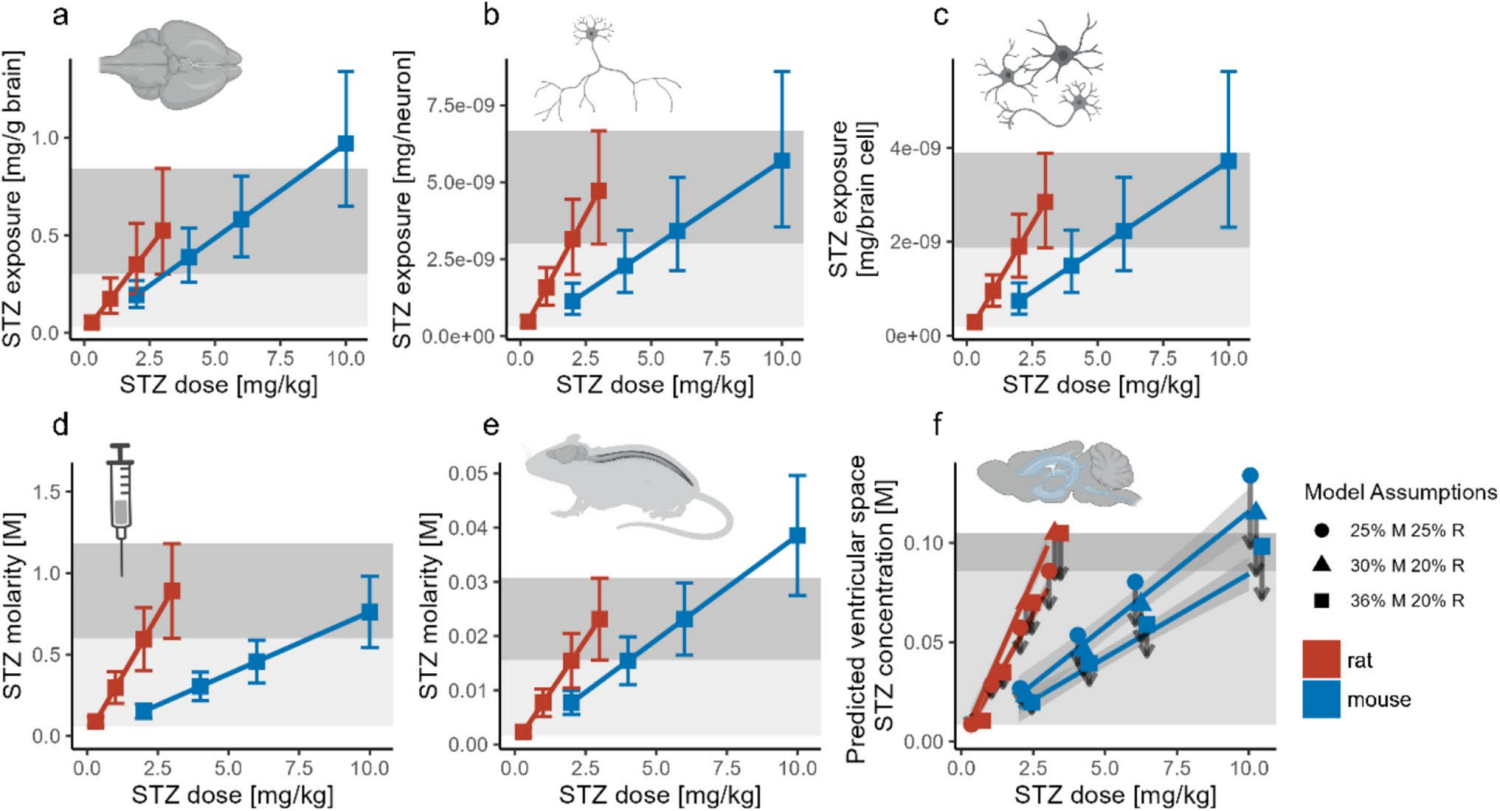
Allometric models showing STZ-icv exposure across different doses in rats and mice. Data points represent 0.3, 1, 2, and 3 mg/kg doses in rats and 2, 4, 6, and 10 mg/kg in mice. The light grey areas represent the range of doses (0.3–3 mg/kg) that consistently induce cognitive deficits in rats. The dark grey area highlights the 3 mg/kg dose, which is commonly used as the standard for inducing cognitive deficits in rats. **a** STZ exposure adjusted for total brain weight, **b** Predicted exposure adjusted for neuronal cell count, **c** Predicted exposure adjusted for both neuronal and non-neuronal cells, **d** Molarity of STZ in the working solution used for STZ-icv modeling in rats and mice, **e** Predicted STZ molarity in CSF, assuming complete and immediate redistribution, **f** Predicted STZ concentration based on ventricular CSF capacity in rats and mice. Black arrows indicate predicted concentrations after 30 min clearance. *CSF* cerebrospinal fluid, *STZ* streptozotocin

Isometric dosing principles that rely on body weight correction can lead to suboptimal STZ exposure in mice, as they overlook the non-proportional relationship between body and brain size in rodents (Herculano-Houzel et al. 2006). To address this, we developed a series of allometric models to estimate STZ exposure in mice comparable to that observed in rats receiving 0.3–3 mg/kg STZ, doses known to consistently induce cognitive deficits in intracerebroventricularly treated rats compared to vehicle-treated controls. We adopted a conservative approach, primarily focusing on the 3 mg/kg dose, despite significant effects being observed even at doses as low as 0.3 mg/kg (Knezovic et al. 2015). The allometric model, which adjusts the STZ dose for brain mass, suggests that a 6 mg/kg dose in mice would achieve an exposure similar to that of 3 mg/kg in rats (Fig. 1a), in line with allometric models used for interspecies dose extrapolation based on body surface area (Nair and Jacob 2016). However, when considering the number of neuronal (Fig. 1b) and total brain cells (Fig. 1c), the 6 mg/kg dose in mice results in a lower average exposure per cell compared to the 3 mg/kg dose in rats. Despite this, the 6 mg/kg dose in mice still covers more than 50% of the predicted exposure range observed with 3 mg/kg in rats, accounting for biological variability in the number of neuronal and non-neuronal brain cells. Next, we calculated the expected average molarity of the STZ solution used for treatment in both rats and mice, assuming an administration of 1 μL/ventricle in mice and 2 μL/ventricle in rats. The molarity of the working solution indicates that achieving an exposure in mice equivalent to 3 mg/kg in rats would require much higher icv doses of 10 mg/kg or more (Fig. 1d). Mice and rats differ in total cerebrospinal fluid (CSF) volume, production, and turnover rates (Rahman et al. 2023). To assess how these differences impact STZ exposure, we first conducted a rough estimation by adjusting exposure based on total CSF volume, assuming absolute and immediate redistribution. This initial model suggested comparable exposure between rats receiving 3 mg/kg and mice receiving 6 mg/kg (Fig. 1e).

Next, we examined how variations in CSF distribution—also influenced by the brain-to-body weight ratio—affect predicted exposure. Mice have a brain-to-body weight ratio approximately 80% higher than rats; however, there is no definitive literature directly comparing CSF distribution in both species using fully comparable methodologies. To account for this uncertainty, we developed three scenarios: i) The ventricular CSF compartment constitutes ~ 25% of total CSF volume in both mice and rats, similar to the estimated relative capacity in humans (Johanson et al. 2008); ii) Due to differences in brain-to-body weight ratios, the ventricular system comprises ~ 30% of total CSF volume in mice and ~ 25% in rats; iii) The ventricular system accounts for ~ 36% of total CSF volume in mice, while remaining at ~ 25% in rats. Next, we used the predicted CSF flow rates for mice and rats, assuming that STZ would be primarily cleared from the ventricular space via the CSF system. This allowed us to estimate how the concentration directly affecting the brain would change within the first 30 min after administration. According to this model, rats receiving 3 mg/kg STZ exhibited concentrations comparable to those in mice treated with 10 mg/kg. However, while this approach provides informative insights, it should be interpreted with caution, as CSF flow is unlikely to be the primary route of STZ clearance. Additionally, in vivo CSF dynamics likely differ significantly from the model scenario due to flow alterations caused by intracerebroventricular injection and potential needle-induced tissue damage (Fig. 1f). Data used for calculation of the allometric model estimates is provided in Supplementary Table 1.

### Isometric dosing does not fully explain the lack of efficacy of STZ-icv in inducing cognitive deficits in C57BL/6 mice

**Fig. 2:**
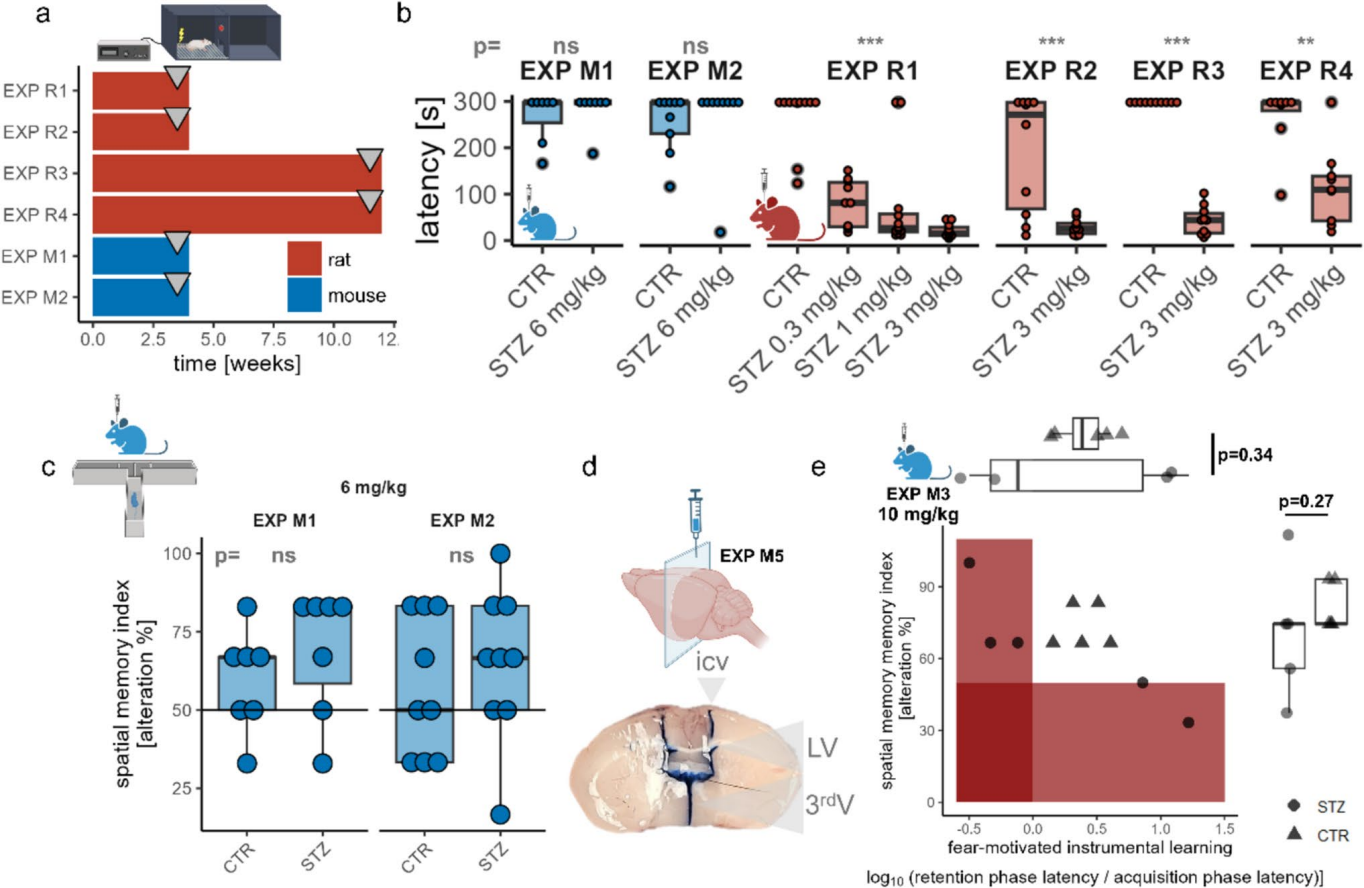
Behavioral experiments showing C57BL/6 mice’s resilience to STZ-icv at doses that induce cognitive deficits in Wistar Han rats. **a** Overview of passive avoidance experiments in the rat and mouse cohorts, **b** Latency in the passive avoidance task across six cohorts, demonstrating reliable cognitive deficits in rats but not in mice, **c** T-maze test confirming the absence of cognitive impairment in mice, assessing spatial memory, **d** Intracerebroventricular methylene blue administration verifying successful STZ delivery to the mouse lateral ventricles, **e** Additional experiment indicating subtle cognitive deficits in female C57BL/6 mice treated with 10 mg/kg STZ. Group comparisons and corresponding p-values were derived from linear models, using the behavioral readout as the dependent variable and treatment, experiment, and their interaction as independent predictors (ns = p > 0.05; * = p < 0.05; ** = p < 0.01; *** = p < 0.001). *CTR* control, *STZ* streptozotocin, *LV* lateral ventricle, *3*^*rd*^*V* third ventricle

We next compared the cognitive effects of STZ-icv in 3-month-old male Wistar rats and 3-month-old male C57BL/6 mice. To evaluate cognitive performance, we used a passive avoidance (PA) task, which measures fear-motivated instrumental learning and is effective in both rats and mice. In accordance with the 3R principles, animals from separate cohorts were used, ensuring all were subjected to identical vehicle (citrate buffer) and STZ procedures and comparable conditions (Fig. 2a). Behavioral testing was performed either 4 weeks after STZ-icv model induction (EXP R1, EXP R2, EXP M1, EXP M2), corresponding to the acute phase of cognitive decline, or after 12 weeks (EXP R3, EXP R4), representing the less pronounced early stage of chronic progressive cognitive decline in rats (Knezovic et al. 2015). Results from the early stage of chronic progressive cognitive decline were included as an additional control to highlight the severity of cognitive impairments in rats compared to their absence in mice. We focused primarily on the 3 mg/kg dose standardly used in rats, but we also included the results from our previous study (EXP R1) with a dose–response for comparison (Knezovic et al. 2015).

As expected, Wistar rats treated with 3 mg/kg STZ-icv exhibited pronounced cognitive deficits (Fig. 2b). Unlike rats given 3 mg/kg STZ-icv, C57BL/6 mice receiving 6 mg/kg STZ-icv, a dose calculated to produce similar drug exposure, did not exhibit cognitive decline within the same timeframe (Fig. 2b). To confirm that the absence of cognitive deficits in mice was not specific to fear-motivated instrumental learning, we subjected STZ-icv-treated C57BL/6 mice to additional behavioral testing. In the spatial memory T-maze task (Fig. 2c), C57BL/6 mice from both cohorts similarly showed no signs of cognitive impairment, further supporting the hypothesis of reduced sensitivity to STZ-icv in comparison to Wistar rats. In C57BL/6 mice, using a comparable experimental setup, we previously observed no cognitive impairments in the novel object recognition and Morris Water Maze tasks (data not shown), a finding supported by the current results.

Of note, the mice used in EXP M1 were purchased from Charles River Laboratories (Sulzfeld, Germany), while those used in EXP M2 were of the same strain but bred in-house at the Croatian Institute for Brain Research. This ruled out potential residual bias arising from factors such as inherent differences in the facility-related microbiome (Ericsson et al. 2021) or substrain variations (Bryant 2011; Mekada et al. 2009; Bryant et al. 2008). Furthermore, different batches of STZ were used in EXP M1 and EXP M2, and two other experiments of similar design, where cognitive deficits were also absent (data not shown), ruling out batch-effect factors.

As a standard validation step for icv injections in mice, we administered 1% methylene blue (MB, EXP M5) using the STZ-icv delivery protocol to ensure accurate STZ delivery to the brain ventricles (Taylor et al. 2021; Redrobe et al. 2002). This confirmed that the lack of cognitive deficits was not due to unsuccessful STZ delivery, ensuring that the icv procedure adapted from rats to mice effectively targeted the same anatomical space (Fig. 2d).

While C57BL/6 mice are less sensitive to STZ-icv-induced neurodegeneration than Wistar rats, they are not entirely resistant. To illustrate this, we included a control experiment where C57BL/6 mice were treated with a 10 mg/kg STZ-icv dose to achieve high exposure (EXP M3). Interestingly, these mice still did not exhibit clear evidence of cognitive deficits in either fear-motivated instrumental learning or spatial working memory. However, when considering the results of both tests, mice treated with STZ-icv showed cognitive decline in at least one of the tests, whereas control animals consistently demonstrated no pathology in either test. This suggests that, at a dose of 10 mg/kg, mice displayed subtle indications of reduced functional cognitive capacity (Fig. 2e). It is important to emphasize that EXP M3 was conducted in female mice. Previous studies have shown that male rats are more sensitive to the effects of STZ-icv than females (Bao et al. 2017). Consequently, if this experiment had been performed in male mice, it might have resulted in a more pronounced cognitive deficit. However, the primary goal of this experiment was not to assess sensitivity levels but to confirm that C57BL/6 mice are not entirely resistant to STZ-icv, which it successfully demonstrated. Given that other experiments were conducted to compare the sensitivity of C57BL/6 mice and Wistar rats to STZ-icv, this experiment was not repeated to adhere to ethical principles and the 3R framework.

### The diminished effectiveness of STZ-icv in inducing cognitive deficits in C57BL/6 mice is evident in its reduced capacity to trigger reactive astrogliosis

**Fig. 3:**
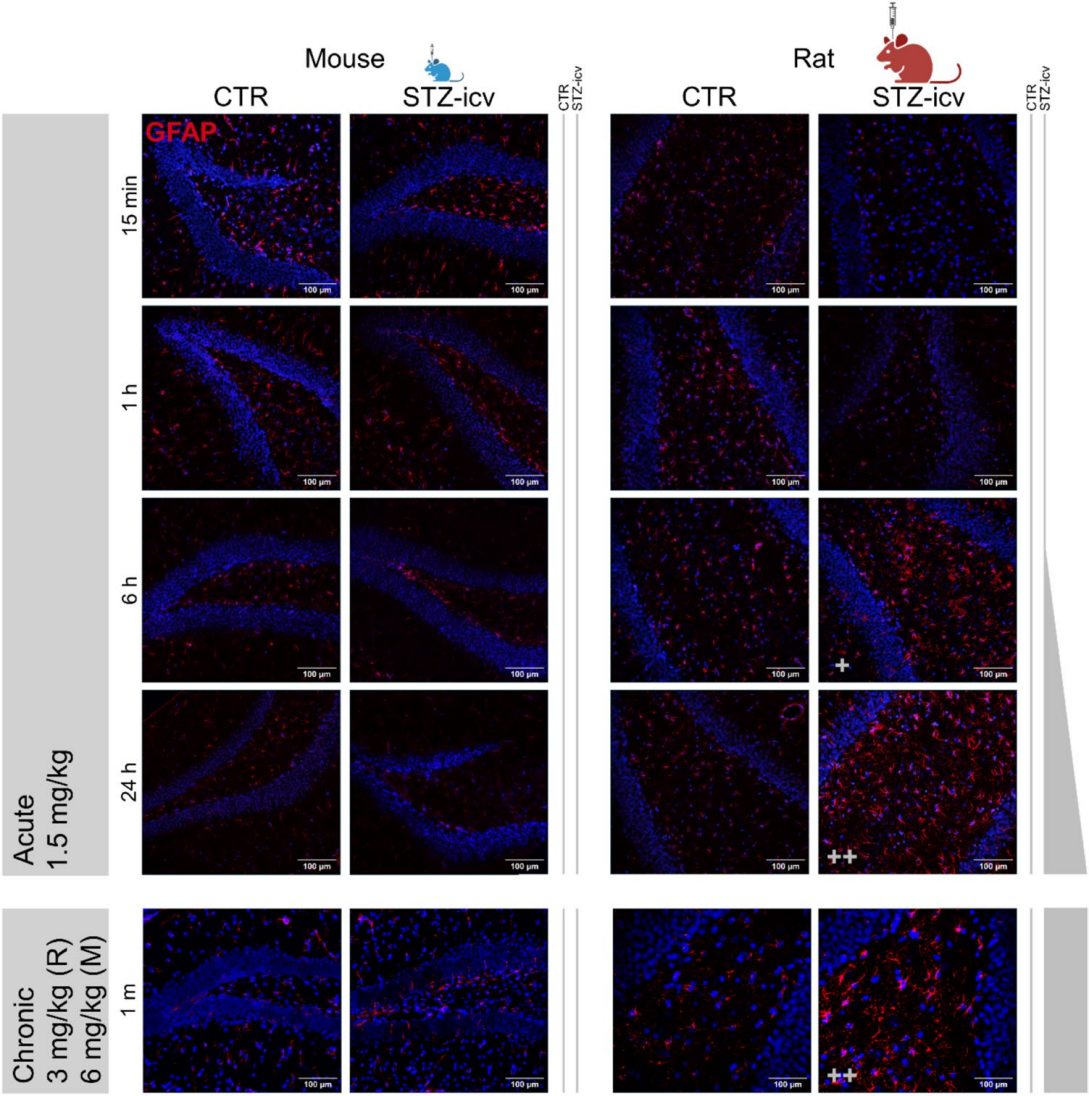
Immunohistochemical analysis of the astrogliotic response to STZ-icv in the hippocampus of mice and rats, assessed within the first 24 h after model induction and at one month—a time point when neuroinflammation and cognitive deficits are typically observed. An increased astrogliotic response, indicated by enhanced immunofluorescent signaling of glial fibrillary acidic protein (GFAP) and denoted by “+”, is evident in rats as early as six hours post-icv treatment and remains present at the one-month mark. In contrast, the changes observed in the mouse hippocampus are minimal and do not exhibit clear signs of reactive astrogliosis. *M* mouse, *R* rat

The significant differences in response to STZ-icv cannot be attributed solely to STZ exposure, especially considering that allometric models indicated that all modelled mouse doses (2, 4, 6, and 10 mg/kg) resulted in greater exposure than 0.3 mg/kg in rats, a dose that still leads to cognitive deficits in rats (Knezovic et al. 2015). To verify that C57BL/6 mice exhibit reduced sensitivity to STZ-icv, we performed an immunohistochemical analysis of GFAP, a marker of reactive astrogliosis (EXP R6, M1, and M4). GFAP upregulation is one of the most consistent pathological changes in AD and is considered a promising biomarker (Askenazi et al. 2023; Grande 2025; Serrano-Pozo et al. 2024; Bellaver et al. 2023). Notably, increased GFAP expression is also a well-established feature of STZ-icv models of AD (Salkovic-Petrisic and Kostrzewa 2021; Knezovic et al. 2017; Mishra et al. 2018). Its upregulation occurs almost immediately after STZ-icv administration (Knezovic et al. 2017), follows a clear dose-dependent pattern, and correlates positively with cognitive deficits in this model (Knezovic et al. 2015; Knezovic and Salkovic-Petrisic 2025). Moreover, GFAP expression is elevated in mice after the successful induction of cognitive deficits with STZ-icv (Fan et al. 2022). This phenomenon appears to be closely linked to the neurotoxic effects of STZ across genetically diverse backgrounds (Chen et al. 2014) and even in different species, including non-human primates (Yeo et al. 2015). Consistent with previous findings (Knezovic et al. 2017; Knezovic and Salkovic-Petrisic 2025), even low doses of STZ-icv induce reactive astrogliosis in the HPC of Wistar rats (Fig. 3). In contrast, C57BL/6 mice treated with 1.5 mg/kg acutely did not exhibit an increase in GFAP (Fig. 3). A similar outcome was observed four weeks after STZ-icv administration, a time point at which cognitive deficits are typically evident in rats, even at doses achieving comparable exposure based on allometric models (Fig. 3).

### A proteomics-based pipeline for identifying pathways underlying species-specific sensitivity to STZ-icv

**Fig. 4:**
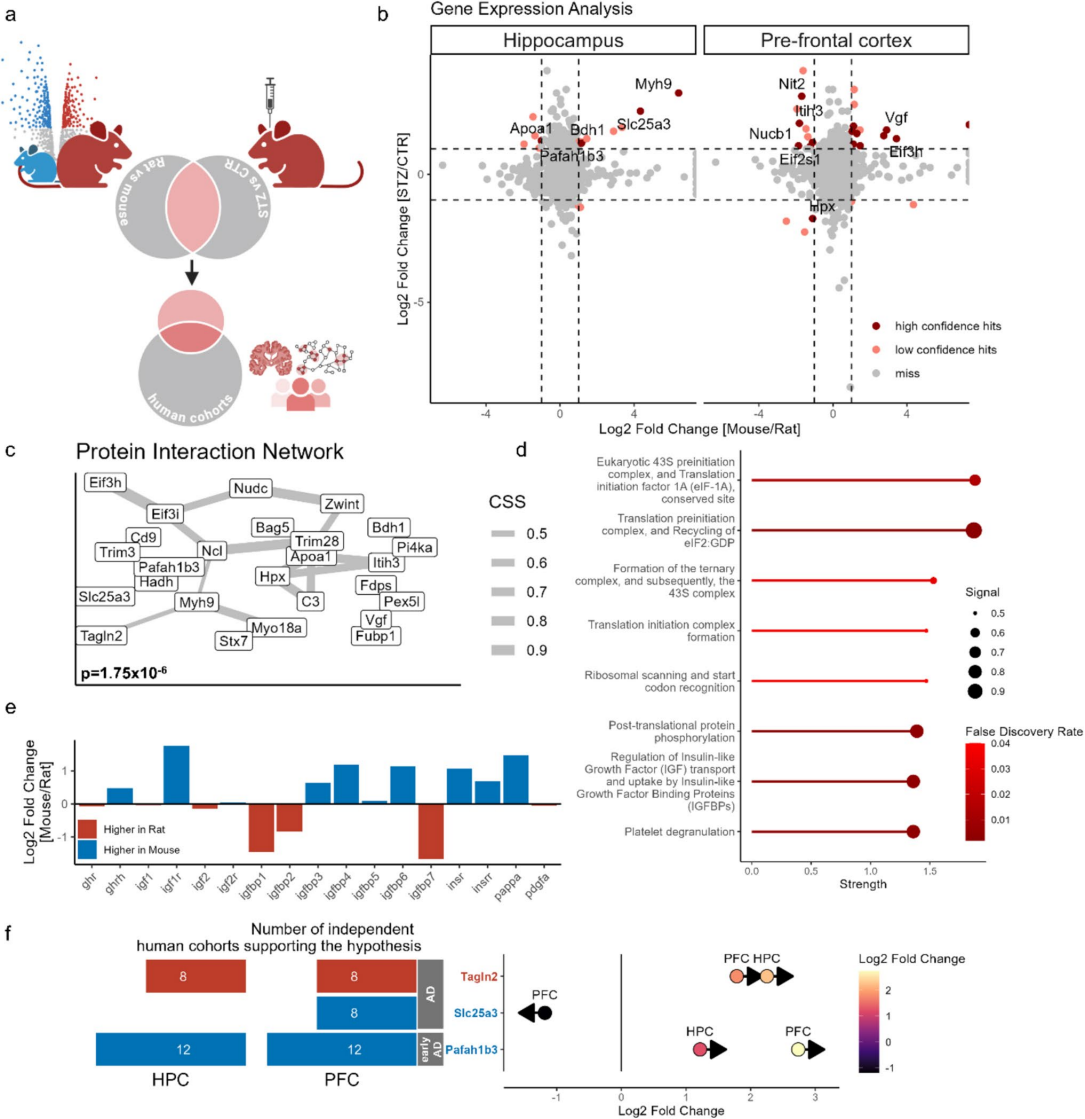
Bioinformatic analysis identifying potential molecular pathways underlying C57BL/6 mice’s resistance to STZ-icv-induced cognitive deficits. **a** Schematic of the approach: identifying differentially expressed genes/proteins in Wistar rats and C57BL/6 mice and comparing them with those altered in human Alzheimer’s disease cohorts, **b** Volcano plot showing differentially expressed genes/proteins in Wistar rats vs. C57BL/6 mice and in the hippocampus and prefrontal cortex of STZ-icv-treated rats, **c** Protein interaction network of candidate proteins linked to STZ-icv susceptibility or resilience, **d** Pathway enrichment analysis of the identified protein network, with confidence levels for each pathway, **e** Differential expression of genes related to insulin-like growth factor (IGF) signalling in Wistar rats and C57BL/6 mice, **f** Candidate proteins also consistently altered in independent human AD cohorts, with the number of confirming studies shown in red (highly expressed in rats) and blue (highly expressed in mice). Arrows indicate the direction of change in both the STZ-icv model and AD patients. *HPC* hippocampus, *PFC* prefrontal cortex, *AD* Alzheimer’s disease

To investigate the reduced sensitivity of C57BL/6 mice to STZ-icv, we developed a pipeline to identify molecular pathways that may account for the differing sensitivity between Wistar rats and C57BL/6 mice (Fig. 4a). We identified proteins with differential abundance between STZ-icv-treated adult male Wistar rats and their respective controls, as reported in the study by da Silva et al. (Silva et al. 2024), and compared them to genes differentially expressed between Wistar rats and C57BL/6 mice, extracted from an RNA-Seq atlas of gene expression in normal mouse and rat tissues (Söllner et al. 2017). Overlapping genes were considered candidate STZ-icv response genes, potentially responsible for the differing sensitivity of Wistar rats and C57BL/6 mice to STZ-icv, as they may mediate the cognitive effects of STZ-icv while being subject to unique species-specific regulation. This approach identified 34 unique genes that are both up- or downregulated by STZ-icv in HPC and/or PFC and are expressed at different levels in mouse and rat brains (Fig. 4b; Supplementary Table 2).

The genes were then analyzed in STRING to identify functional protein interaction networks, utilizing data from experimentally determined interactions, curated databases, predicted interactions based on gene neighborhood, gene fusions, co-occurrence, co-expression, homology, and co-mention in published literature (Szklarczyk et al. 2023). The original network consisted of 34 nodes (candidate genes) and 19 edges, with an average node degree of 1.12 and an average local clustering coefficient of 0.338. Given the expected number of edges, the network was determined to be significantly enriched for interactions (p = 8.19 × 10^–5^). Functional analysis was performed on both the original and expanded networks to provide additional insights into the identified pathways (Fig. 4c). Local network clustering and Reactome pathway analysis of the protein interaction network (with five additional populated nodes; p = 1.75 × 10^–6^) identified several signaling pathways as potential STZ-targeted mechanisms underlying the increased resistance of C57BL/6 mice compared to Wistar rats to STZ-icv. Notably, several closely related pathways highlighted the regulation of translation initiation as a potential key pathway uniquely affected by STZ-icv in mice and rats (Fig. 4d).

Another intriguing pathway identified was the regulation of insulin-like growth factor (IGF) transport and uptake, aligning with the long-standing hypothesis that the STZ-icv model mimics human sAD by disrupting brain insulin signaling—a central feature of molecular pathways implicated in AD (Alves et al. 2021). Based on the identification of this pathway, we selectively extracted data on species-specific brain expression of individual proteins from the IGF-1 protein interaction network within STRING (Fig. 4e), observing differences that may provide valuable insights not only for the STZ-icv model but also for other rodent models of AD, given the critical role of insulin signaling in disease etiopathogenesis. For instance, while the expression levels of IGF-1 appear similar in both species, mice exhibit higher expression of both IGF-1 and insulin receptors in the brain (Fig. 4e).

Finally, we queried the NeuroPro database with a list of candidate genes that are differentially expressed in the brains of mice and rats and encode proteins that show varying abundance in the brains of STZ-icv treated animals. Our focus was on proteins with consistent patterns of differential expression across at least five independent human studies, matching the direction of changes observed in the STZ-icv model. This approach led us to narrow down to three key genes: *Tagln2*, *Slc25a3*, and *Pafah1b3*. Among these, *Tagln2* was the only gene found at higher levels in rat brains compared to mice. *Tagln2* encodes transgelin 2, which has been found to be consistently elevated in both the HPC and PFC of AD patients (Fig. 4f) (Askenazi et al. 2023). Although transgelin 2 is predicted to have actin-binding activity, its exact physiological and pathophysiological functions remain poorly understood. The other two genes, *Slc25a3* and *Pafah1b3*, are expressed at higher levels in the brains of mice compared to rats. *Slc25a3* has been consistently found to be downregulated in the PFC of AD patients, while *Pafah1b3* is the only gene among the three identified that encodes a protein increased in both the HPC and PFC during the early stages of AD. These three poorly characterized genes/proteins appear to play significant roles in AD pathogenesis. The fact that their expression is altered in the STZ-icv model in a manner consistent with changes observed in human AD suggests that this model could offer valuable insights into their physiological and pathological roles.

### Validation and expansion of the bioinformatics approach through transcriptomic analysis

**Fig. 5:**
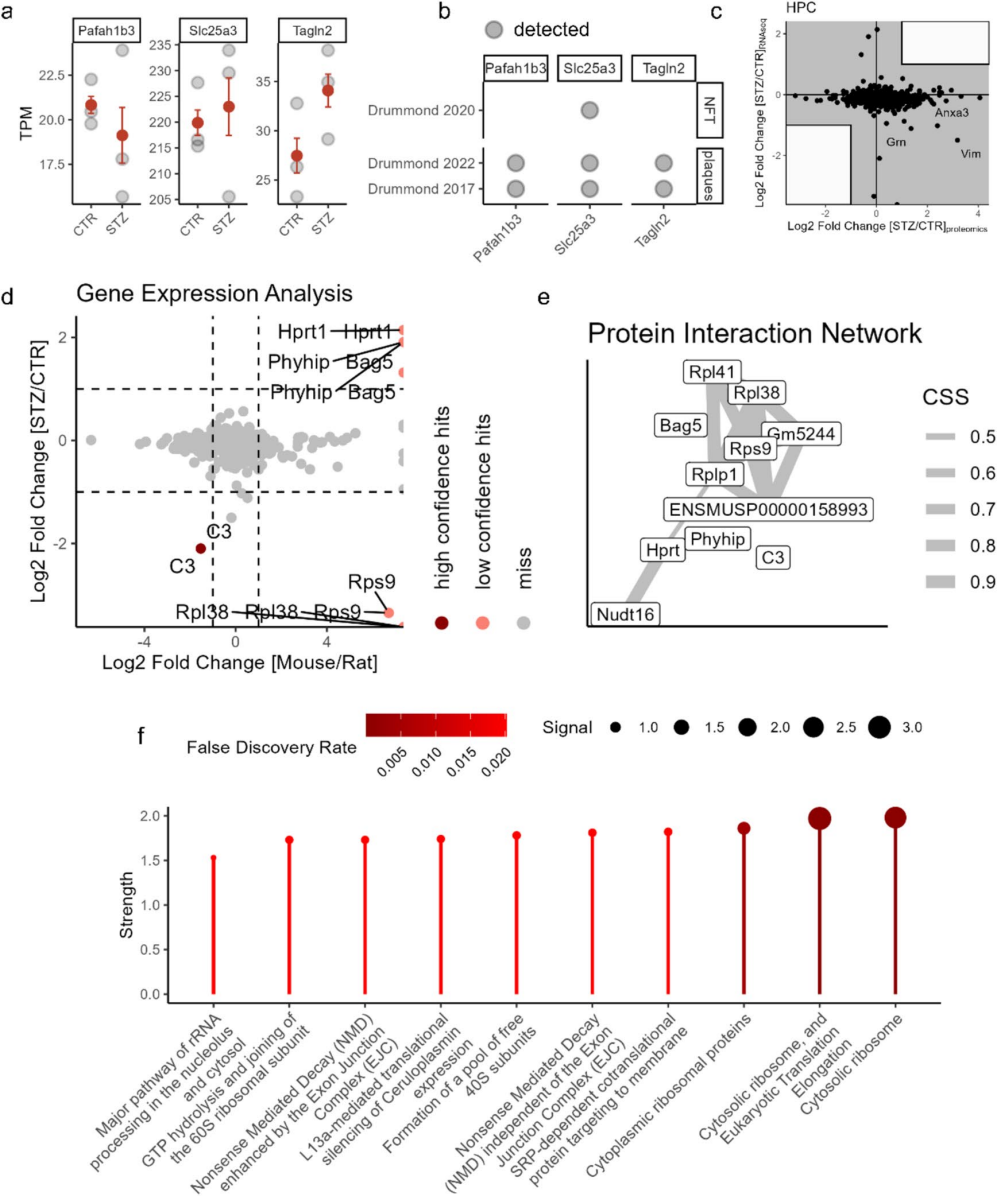
Validation and enhancement of the bioinformatics approach through transcriptomic analysis. **a** Expression levels of three proteins identified using a pipeline based on proteomic changes in STZ-icv rats, **b** Identification of Pafah1b3, Slc25a3, and Tagln2 in proteomic datasets from plaques and tangles in AD brains, **c** Correlation of differential gene expression and differential protein abundance from the merged and curated STZ-icv HPC dataset, **d** Volcano plot showing differentially expressed genes/proteins in Wistar rats vs. C57BL/6 mice, and in the hippocampus and prefrontal cortex of STZ-icv-treated rats, **e** Protein interaction network of candidate proteins linked to STZ-icv susceptibility or resilience, **f** Pathway enrichment analysis of the identified protein network, with confidence levels for each pathway. *HPC* hippocampus, *AD* Alzheimer’s disease, *STZ* streptozotocin

The initial step of our bioinformatics pipeline was based on the proteomic data provided by da Silva et al. (Silva et al. 2024). To further investigate the changes in the three identified proteins in both AD and the STZ-icv model, we analyzed the transcriptomic profiles of their respective genes to uncover the potential causes of their altered abundance. For this analysis, we employed RNA sequencing data from the study by Fine et al. (Fine et al. 2024). Examination of normalized transcript counts from the reanalyzed raw data shows that, for the three proteins identified as differentially abundant, none exhibited transcript changes in the same direction as observed at the protein level (Fig. 5a). Notably, Tagln2 showed a trend toward increased expression, although this did not reach statistical significance. These findings suggest that the observed proteomic alterations are unlikely to be driven by transcriptional regulation (Fig. 5a).

The increased presence of Pafah1b3 and Tagln2, despite their lack of increased expression at the transcriptomic level, may be attributed to a reduced rate of their removal. One possible explanation is that these proteins accumulate in plaques and/or neurofibrillary tangles (NFTs), which impedes their efficient degradation or removal. To investigate this hypothesis, we combined data from three proteomic studies focused on proteins found in plaques and NFTs in the brain tissue of AD patients (Drummond et al. 2017; Drummond et al. 2022; Drummond et al. 2020). Notably, both Pafah1b3 and Tagln2 were identified as components of plaques in AD, while Slc25a3 was detected not only in plaques but also in NFTs (Fig. 5b).

The increased differential abundance of proteins, potentially driven by deposition rather than upregulation of their respective genes, could, in theory, obscure the detection of some key pathways in our pipeline. To test this, we next examined the correlation of log2-fold changes for all detected genes/proteins in the HPC across both RNAseq and proteomic datasets, focusing specifically on genes/proteins with log2-fold changes of <−1 or > 1 in both datasets (Fig. 5c). We did not observe a clear correlation, and no genes passed this stringent threshold, suggesting that our pipeline could benefit from a separate approach focused on detecting candidate genes/proteins at the transcriptomic level. To further investigate, we expanded our analysis using the same method previously applied to the proteomics dataset. By focusing on the most differentially expressed genes in both mice and rats, which overlap with those differentially expressed between STZ-icv and control rats, we identified a protein interaction network that again pointed to STZ-icv candidate pathways being linked to ribosomal proteins and translation-related processes (Fig. 5d, e).

### Identification of AD-related candidate pathways contributing to the susceptibility of Wistar rats to STZ-icv

**Fig. 6:**
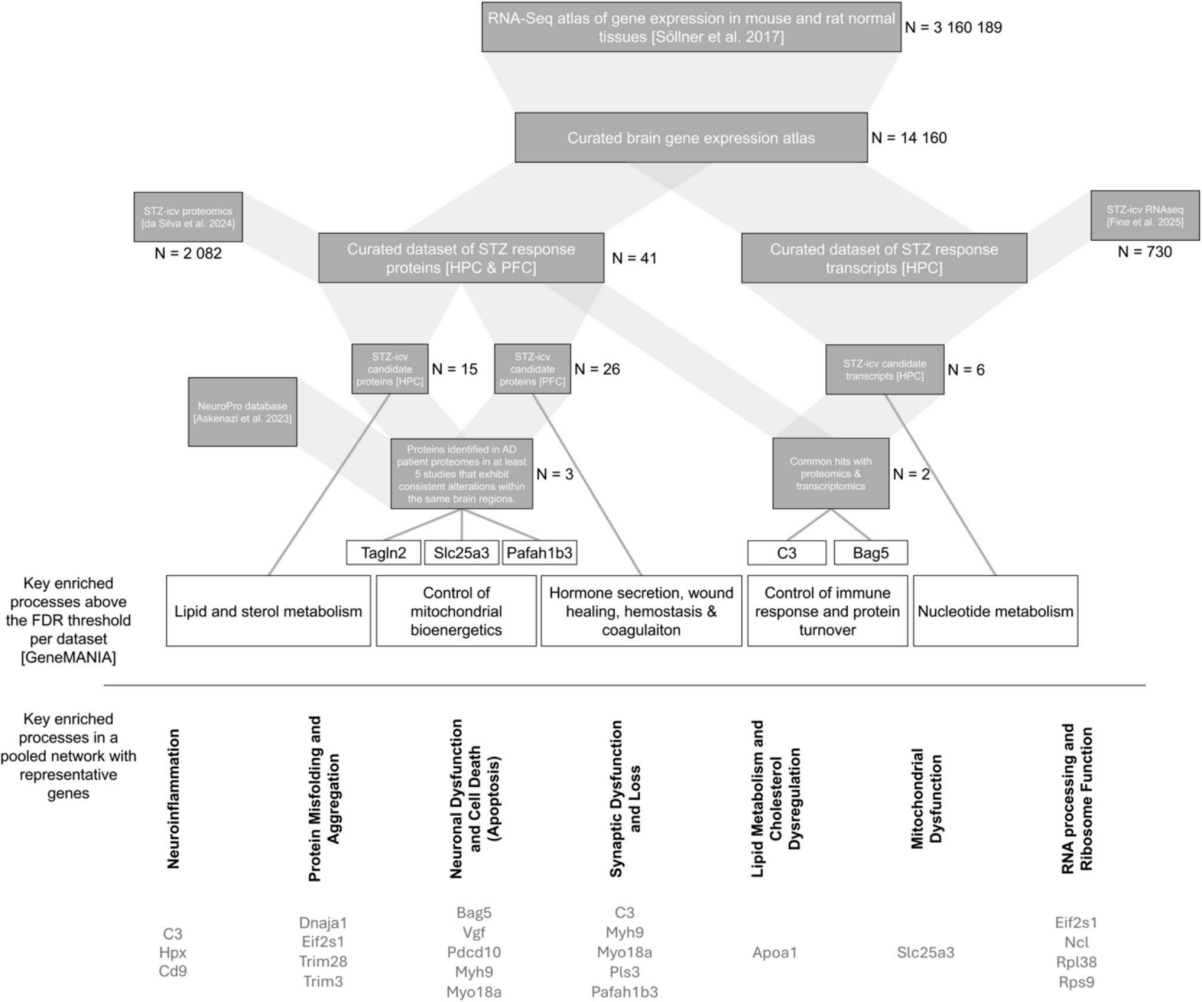
Schematic overview of the pipeline used to identify STZ-icv candidate pathways based on species-specific sensitivity to STZ-icv. The number of hits in each curated dataset is indicated by “N”

An integrative analysis of protein interaction networks, derived from the proteomic changes reported in STZ-icv and those inferred from RNAseq data, reveals that ribosome biogenesis and function, translation initiation, elongation, and regulation, as well as mRNA processing and surveillance, may be key pathophysiological pathways driving STZ-icv-induced neurodegeneration and cognitive decline. A closer examination of curated databases further highlights specific pathways enriched in particular datasets (Fig. 6). For instance, using GeneMANIA algorithms, we identified an enrichment of lipid and sterol metabolism pathways in the hippocampus of STZ-icv-treated rats, and an enrichment of hormone secretion, wound healing, hemostasis, and coagulation regulatory pathways in the prefrontal cortex of STZ-icv-treated rats, after focusing on pathways that are differentially regulated between mice and rats.

The most stringent analysis, where candidate proteins were filtered to retain only those enriched in human proteomic studies of AD patients and altered in the same brain regions in the same direction in both STZ-icv rats and AD patients, revealed an enrichment of pathways involved in the regulation of mitochondrial bioenergetics. Common proteins found in the protein interaction networks from both proteomic and RNAseq-based approaches include C3 and Bag5, suggesting that the control of innate immune response and protein turnover are key processes identified across independent approaches utilizing different datasets.

Finally, by specifically focusing on the network derived from RNAseq data filtered for species-specific pathways, we identified nucleotide metabolism pathways as being involved in the effects of STZ-icv in the hippocampus. The pooling of proteins from these identified pathways suggests that STZ-icv induces neurodegeneration and cognitive dysfunction by impacting multiple pathways recognized as key pathophysiological factors in the development of AD, including neuroinflammation, protein misfolding and aggregation, neuronal dysfunction and apoptosis, synaptic dysfunction and loss, lipid metabolism, mitochondrial dysfunction, and RNA processing and ribosome function.

## Discussion

In this study, we examine the species-specific sensitivity to STZ-icv in two commonly used strains for modeling sAD: male Wistar Han rats and male C57BL/6 mice. Although allometric scaling suggests that mice are often underdosed in STZ-icv experiments compared to rats treated with 3 mg/kg, this factor alone does not fully account for the lack of efficacy of STZ-icv in inducing cognitive deficits in C57BL/6 mice (Fig. 1). We confirmed this by showing that C57BL/6 mice do not exhibit cognitive deficits following STZ-icv administration, even at doses that result in similar exposure levels to those that clearly cause significant cognitive deficits in Wistar rats (Fig. 2). Additionally, we further confirm the lack of efficacy of STZ in C57BL/6 mice at the molecular level by analyzing reactive astrogliosis through GFAP immunohistochemistry, a reliable marker of neurodegeneration in both STZ-icv models and AD patients (Fig. 3). After ruling out other potential contributors, including possible subtle genetic differences and other facility-related confounding variables, the precision of STZ-icv administration in mice, STZ batch effects, and the sensitivity of behavioral tests, our findings point to the involvement of additional, as-yet-unknown biological factors that enhance the resilience of C57BL/6 mice to STZ-icv-induced cognitive deficits.

Using a bioinformatics approach, we developed a pipeline to identify key genes, proteins, and pathways that may mediate the effects of STZ-icv on cognition and neurodegenerative processes. Our approach began by utilizing the RNA sequencing atlas for Wistar rats and C57BL/6 mice to identify genes that are differentially expressed in these species (Söllner et al. 2017). We then compared these differentially expressed genes with a dataset containing differentially abundant proteins (Silva et al. 2024) and genes (Fine et al. 2024) that are differentially expressed between STZ-icv and control groups. Beyond serving as a proof of concept for using species-specific sensitivity and multiomics to explore potential mediator pathways, this study offers valuable insights into pathways relevant to the widely used STZ-icv model of sporadic Alzheimer’s disease. In this discussion, we highlight these findings, starting with broader observations and then focusing on more specific pathophysiological mechanisms that could enhance our understanding of both the STZ-icv model and the development of Alzheimer’s disease.

Independent analysis starting both from the proteomic dataset from da Silva et al. (Silva et al. 2024) and an RNA sequencing study by Fine et al. (Fine et al. 2024) identified protein networks enriched in signaling pathways related to ribosomal proteins and translation-related processes such as translation initiation, strongly enriched in the dataset obtained by analysis of proteomic changes. Dynamic regulation of mRNA translation initiation and elongation is critical for the survival and function of neural cells (Kapur et al. 2017). Dysregulation of translation initiation, whether through mutations in the translational machinery or inappropriate activation of the integrated stress response, is a well-studied pathological mechanism driving neurodegeneration. The link between AD and reduced global translation was first identified in 1989, based on evidence showing that polysomes from the cortices of AD patients contained less RNA compared to those from control subjects (Langstrom et al. 1989). Subsequent studies have reinforced this link, consistently showing aberrant translation initiation as a key pathway affected in AD, both in preclinical in vitro and in vivo models and in post-mortem analyses of brain tissues from AD patients (Kapur et al. 2017; Oliveira and Klann 2022; Ghosh et al. 2020).

Another pathway identified through the aforementioned approach is the regulation of IGF transport and uptake. In the proteomics approach, the involvement of this pathway was directly detected through enrichment (Fig. 4). In contrast, the transcriptomics-based approach showed a weaker and more indirect association, although it still highlighted key elements such as Bag5, an important regulator of Akt (Bracho-Valdés et al. 2023), ribosomal proteins involved in insulin signaling response, and PHYHIP through its interaction with DYRK1 A (Bescond and Rahmani 2005), which plays a role in regulating insulin signaling (Barzowska et al. 2021) (Fig. 5).

The potential involvement of insulin signaling in AD was first suggested in 1983, when Bucht et al. reported changes in blood glucose levels and insulin secretion in AD patients (Bucht et al. 1983). Fifteen years later, Frölich et al. described alterations in neuronal insulin signal transduction pathways in AD brains (Frölich et al. 1998), leading to Hoyer’s proposal that AD could be considered a brain-specific form of non-insulin-dependent diabetes mellitus (Hoyer 1998) (with a later suggestion by de la Monte to name AD diabetes mellitus type 3 (Steen et al. 2005)). Since then, extensive preclinical and clinical research has supported this hypothesis, providing compelling evidence that insulin dysregulation may not only contribute to the progression of the disease but could also be a causative factor, driving the disruption of other pathways implicated in AD (Alves et al. 2021; Kellar and Craft 2020).

Due to their shared evolutionary origin, insulin and IGF signaling in the brain exhibit substantial overlap, sharing not only signaling pathways but also receptors (Kleinridders 2016; Fernandez and Torres-Alemán 2012). Insulin and IGF1 receptors in the brain can heterodimerize to form complexes capable of binding both ligands, albeit with greater affinity for IGF compared to insulin (Kleinridders 2016). It is unsurprising that the insulin/IGF signaling pathway emerged as a significant potential mediator of the effects of STZ-icv, given that the STZ-icv model was originally developed to study insulin dysregulation in the context of sAD and has been used for this purpose for nearly three decades (Salkovic-Petrisic and Kostrzewa 2021; Grünblatt et al. 2007). However, the identification of this pathway as a candidate mechanism underlying the absence of STZ-icv effects in C57BL/6 mice was unexpected.

Upon closer examination of the expression profiles of genes associated with IGF signaling, it is evident that while IGF1 mRNA levels are comparable in the brains of rats and mice, mRNAs encoding IGF1 and insulin receptors are expressed at higher levels in mice than in rats (Fig. 3e). This difference is accompanied by a distinct profile of IGF binding protein (IGFBP) expression: IGFBP1, IGFBP2, and IGFBP7 are more highly expressed in rats, whereas IGFBP3, IGFBP4, and IGFBP6 are expressed at higher levels in mice. Since IGFBPs play a crucial role in regulating IGF availability in the brain without directly affecting insulin levels, it is tempting to hypothesize that brain insulin/IGF signaling differs fundamentally between rats and mice. These differences may align with the already recognized evolutionary variations in insulin regulation between the two species (Shiao et al. 2008). This underscores the potential shortcomings of the common practice of grouping data on insulin/IGF signaling from both species under the generalized category of “rodent” brain insulin signaling. The implications and biological significance of differences in the expression of proteins associated with brain insulin/IGF signaling should be further investigated, particularly in the context of rodent models of AD.

Our bioinformatics pipeline also identified specific candidate mediators of the effects of STZ-icv on cognitive capacity and neurodegeneration. Using the NeuroPro curated database (Askenazi et al. 2023), we investigated whether proteins identified as potential mediators of the STZ-icv response are also consistently observed in human proteomic studies. This analysis allowed us to narrow down to three key genes: *Tagln2*, *Slc25a3*, and *Pafah1b3*. Among these, *Tagln2* was the only gene expressed at higher levels in rat brains compared to mice. *Tagln2* encodes transgelin-2, a protein consistently elevated in HPC and PFC of AD patients (Fig. 3f) (Askenazi et al. 2023), as well as in HPC and PFC of STZ-icv-treated rats (Silva et al. 2024). Although transgelin-2 remains relatively understudied, current evidence suggests it plays a critical role in actin cytoskeleton reorganization (Yin et al. 2019). Furthermore, its unique status as the sole transgelin family member found in leukocytes implies a potential role in immunity and inflammation (Jo et al. 2018).

*Pafah1b3* is the only gene among the three identified that is associated with early AD. Its levels are elevated in both HPC and PFC of STZ-icv-treated rats, as well as in AD patients (Silva et al. 2024; Askenazi et al. 2023; Sung et al. 2023). *Pafah1b3* encodes the platelet-activating factor acetylhydrolase IB subunit gamma, a catalytic subunit that removes an acetyl group from the glycerol backbone. This protein plays a critical role in brain development, has been linked to developmental disorders such as lissencephaly, and may function as a central hub protein in mitogen-activated protein kinase metabolic pathways (Huang et al. 2023). Notably, the loss of its close homolog, *PAFAH1B2*, has been shown to significantly reduce amyloid-beta production in both *Drosophila melanogaster* and human cells (Page et al. 2012), suggesting that Pafah1b3 may contribute to neurodegeneration.

*Slc25a3* is expressed at significantly higher levels in the brains of mice compared to rats. Proteomics reveal that this protein is decreased in PFC of STZ-icv rats relative to controls and is consistently reduced in the PFC of AD patients (Silva et al. 2024; Askenazi et al. 2023). *Slc25a3* encodes a mitochondrial phosphate carrier that facilitates the transport of phosphate ions across the mitochondrial membrane, either through proton cotransport or exchange with hydroxyl ions, to support oxidative phosphorylation (Seifert et al. 2015). As a key regulator of inorganic phosphate availability for oxidative phosphorylation, its depletion directly impairs ATP production from ADP. Furthermore, recent evidence suggests that its role extends beyond mitochondrial bioenergetics, as it appears to function as a negative regulator of inflammasome activation (Xiao et al. 2024). The ~ 20-fold higher expression of *Slc25a3* in mice compared to rats, alongside its marked reduction in STZ-icv rats and AD patients, suggests that the greater *Slc25a3* reserve in mice may contribute to their reduced susceptibility to STZ-icv-induced cognitive deficits. This hypothesis is supported by evidence that an ~ 85% reduction in *Slc25a3* is required to significantly impair oxidative phosphorylation in human cells (Seifert et al. 2016).

The integration of proteomic and transcriptomic data revealed that the increased abundance of neither Pafah1b3 nor Tagln2 is associated with higher expression levels (Fig. 5a). Additionally, the analysis of proteomes from plaques and NFTs in Alzheimer’s disease patients suggests that the elevated presence of these proteins may be due to their deposition in protein aggregates, rather than being driven by pathophysiological processes that increase their expression (Fig. 5b). In this context, their role in the STZ-icv model and AD should be investigated in terms of a potential toxic gain of function in their deposited state within plaques and tangles, as well as the possible toxic effects of their reduced availability due to deposition.

In contrast, the third candidate protein, Slc25a3, is less abundant; however, transcriptomic data suggest its expression is not significantly downregulated. This implies that reduced protein availability may stem from altered turnover or stability rather than diminished expression—a factor potentially relevant to mitochondrial bioenergetics and inflammation. Additionally, Slc25a3 has been identified in both plaques and neurofibrillary tangles. Investigating its upregulation and/or stabilization as a potential pharmacological strategy warrants consideration.

Comparing contributors to the protein interaction networks constructed from independent proteomic and transcriptomic dataset analyses revealed C3 and Bag5 as common proteins identified by both approaches as differentially abundant in STZ-icv rats compared to their respective controls, while also being differentially expressed in both rats and mice. Both proteins appear to play a significant role in pathways associated with AD. C3, a component of the complement system, is involved in AD pathophysiology by regulating microglial activity, neuroinflammation, synaptic function, and amyloid β pathology (Wu et al. 2019). Notably, C3 seems to be a hallmark of sex-dependent AD sensitivity, as decreasing β-estradiol levels trigger S-nitrosylation of C3, which leads to synaptic phagocytosis (Yang et al. 2022). It is possible that a similar process may contribute to sex-dependent sensitivity to STZ, a hypothesis that warrants further experimental investigation (Bao et al. 2017). Bag5 appears to have a protective role in Alzheimer’s disease, as its levels are increased in the brains of AD mice, while its silencing has been shown to exacerbate the toxic effects of amyloid-β and promote the production of reactive oxygen species (Guo et al. 2015; Wang et al. 2014), which are key contributors to AD (Homolak and Çakatay 2022). Our analysis revealed increased Bag5 expression in STZ-icv rats, along with higher protein levels in the prefrontal cortex but lower levels in the hippocampus of STZ-icv-treated mice. Furthermore, gene expression data from healthy rat and mouse brains show that Bag5 expression is significantly higher in mice, suggesting it may act as a protective factor against neuroinflammation in STZ-icv-treated mice.

Building on the observation that C57BL/6 mice appear more resilient to STZ-icv than Wistar rats, this study employs a bioinformatics approach that integrates datasets on normal brain gene expression in both species with curated multiomics data from STZ-icv-treated rats. Through this analysis, we identify key molecular pathways and proteins involved in cognitive deficits and neuroinflammation in STZ-icv rats, which seem to be less affected by STZ-icv in mice. By comparing candidate proteins and protein networks identified through this approach with data from human cohorts, we confirm their relevance to AD and establish a framework for their further investigation in the STZ-icv model as well as in other AD models.

Limitations.

While our study demonstrates the resilience of C57BL/6 mice to STZ-icv-induced cognitive deficits and reactive astrogliosis, other studies have reported successful induction of such deficits and neuroinflammation in the same strain (Nasiri et al. 2020; Wang et al. 2019; Zhang et al. 2023; Yamini et al. 2022; Liu et al. 2022; Qi et al. 2021). The most likely explanation for this discrepancy lies in the variability of STZ-icv protocols across laboratories, as discussed in our previous work (Homolak et al. 2021). For instance, some groups utilize stereotaxic navigation and an infusion pump to deliver STZ over several minutes (Moreira-Silva et al. 2019). In certain protocols, the administration process alone (excluding animal preparation and instrument setup) takes up to 13 minutes (Poddar et al. 2020). While these methods provide more precise spatiotemporal control over STZ dosing, they also introduce potential confounding factors, such as STZ anomer equilibration (which may affect toxin potency), increased physical brain damage, and prolonged anesthesia—all of which could influence the extent to which STZ induces cognitive deficits. One of the key strengths of our study is that we maintained identical experimental conditions for both mice and rats, ensuring consistency in chemical batches and procedural execution. This approach allowed for a direct comparison between species. In contrast, other studies typically focus on either mice or rats, limiting their ability to make direct cross-species comparisons. While we acknowledge that STZ-icv may have an effect in C57BL/6 mice, as evidenced by the experiment using a 10 mg/kg dose, our findings suggest a reduced potency in this strain, as shown by our direct comparison of its effects in both species. Definitive confirmation will require replication of our results by other laboratories using their own adapted protocols in both species simultaneously.

Using an immunohistochemical approach, we demonstrated that STZ-icv does not elicit either acute or chronic astrogliotic responses in C57BL/6 mice. In the acute experiment, we compared the effects of a 1.5 mg/kg dose in both mice and rats. This dose was chosen to specifically evaluate the immediate effects of STZ, as the standard induction protocol involves two administrations of 1.5 mg/kg, spaced 48 h apart (3 mg/kg in total). Our approach mimicked the initial dose, which is already sufficient to provoke a neuropathological response. Under these conditions, a pronounced astrogliotic reaction was observed in STZ-icv-treated rats, whereas no such response was evident in mice. Due to ethical considerations, it was not feasible to introduce additional dose groups that might have allowed a more precise comparison of STZ-icv potency between the two species—especially in the context of doses predicted by various allometric scaling models. Nonetheless, our immunohistochemical analysis of GFAP in chronic experiments, using 3 mg/kg for rats and 6 mg/kg for mice, confirmed that the GFAP response was absent or markedly reduced in mice, even at doses expected to yield comparable exposure based on allometric predictions.

One limitation of the approach described in this study is its reliance on reanalyzing datasets from other research groups due to the lack of more suitable data. While we view this as a strength and an approach aligned with the 3R principles, a potential drawback is that minor methodological differences between experiments may introduce bias. For instance, as highlighted in Table 1, the proteomics dataset obtained from da Silva et al. was based on tissue from Wistar rats treated with 3 mg/kg STZ-icv and sacrificed two weeks after model induction. In contrast, the study by Fine et al. used Long-Evans rats treated with 4.5 mg/kg STZ-icv, with euthanasia occurring six weeks post-treatment. While these methodological differences can provide support for findings that remain consistent across multiple datasets they may also reduce the power to detect certain changes by introducing variability. This may, in part, explain the absence of correlation observed between proteomic and transcriptomic data from STZ-icv rats in this study (Fig. 5c), although this could also be attributed to the well-documented weak correlation between gene expression and protein abundance reported in the literature (Vogel and Marcotte 2012). To address this, we leveraged these differences as an opportunity to refine our pipeline—not by dismissing discrepancies between datasets but by exploring them as potential overlooked targets to enhance sensitivity. Nonetheless, methodological variations in the original studies from which raw data were obtained should always be carefully considered.

## Conclusion

In this study, we explore the potential factors underlying the observed resilience of C57BL/6 mice to STZ-icv-induced cognitive deficits and reactive gliosis. Initially, we examine methodological factors that could result in underexposure of mouse neurons to STZ, taking into account anatomical and physiological differences between species. As the allometric models do not provide a clear explanation for the absence of effects in C57BL/6 mice, we conducted further investigations focusing on the differential abundance of proteins and differential expression of genes in STZ-icv-treated rats and their respective controls, as well as the differentially expressed genes in the brains of Wistar Han rats compared to C57BL/6 mice. This enabled us to identify key signaling pathways that may contribute to the resilience of C57BL/6 mice to STZ-icv-induced cognitive deficits, with a particular emphasis on pathways related to ribosomal proteins and translation-related processes, such as translation initiation. The methodology we employed serves as a proof-of-concept for a pipeline that could be used to identify potential druggable disease-related pathways based on strain- or species-specific differences. Additionally, our research highlights three key proteins (Tagln2, Slc25a3, and Pafah1b3) that are differentially abundant in Wistar Han rats and C57BL/6 mice, are affected by STZ-icv in the model that develops cognitive deficits and are also consistently altered in the same direction across human AD cohorts. The identified proteins, warrant further investigation and experimental validation, along with exploration of their potential as pharmacological targets for AD.

## Supplementary Information

Below is the link to the electronic supplementary material.

Supplementary file1 (XLSX 14 KB)

Supplementary file2 (XLSX 18 KB)

## Data Availability

The raw data for allometric models is available in Supplementary Table 1, while the raw data for each candidate gene can be found in Supplementary Table 2. Additional data not included in the supplements can be requested from the corresponding author.
